# Overexpression of *PAD1* and *FDC1* results in significant cinnamic acid decarboxylase activity in *Saccharomyces cerevisiae*

**DOI:** 10.1186/s13568-015-0103-x

**Published:** 2015-02-18

**Authors:** Peter Richard, Kaarina Viljanen, Merja Penttilä

**Affiliations:** VTT Technical Research Centre of Finland Ltd, Tietotie 2, P.O. box 1000, 02044 VTT Espoo, Finland

**Keywords:** Cinnamic acid, Styrene, Decarboxylase, *S. cerevisiae*, *PAD1*, *FDC1*

## Abstract

The *S. cerevisiae PAD1* gene had been suggested to code for a cinnamic acid decarboxylase, converting *trans*-cinnamic acid to styrene. This was suggested for the reason that the over-expression of *PAD1* resulted in increased tolerance toward cinnamic acid, up to 0.6 mM. We show that by over-expression of the *PAD1* together with the *FDC1* the cinnamic acid decarboxylase activity can be increased significantly. The strain over-expressing *PAD1* and *FDC1* tolerated cinnamic acid concentrations up to 10 mM. The cooperation of Pad1p and Fdc1p is surprising since the *PAD1* has a mitochondrial targeting sequence and the *FDC1* codes for a cytosolic protein. The cinnamic acid decarboxylase activity was also seen in the cell free extract. The activity was 0.019 μmol per minute and mg of extracted protein. The overexpression of *PAD1* and *FDC1* resulted also in increased activity with the hydroxycinnamic acids ferulic acid, p-coumaric acid and caffeinic acid. This activity was not seen when *FDC1* was overexpressed alone.

An efficient cinnamic acid decarboxylase is valuable for the genetic engineering of yeast strains producing styrene. Styrene can be produced from endogenously produced L-phenylalanine which is converted by a phenylalanine ammonia lyase to cinnamic acid and then by a decarboxylase to styrene.

## Introduction

Enzymes for the decarboxylation of hydroxycinnamic acids such as p-coumaric acid, caffeic acid or ferulic acid have been described. A ferulic acid decarboxylase and the corresponding gene, *fdc*, was identified from *Bacillus pumilus* (Zago et al. 1995). A ferulic acid decarboxylase was also identified from an *Enterobacter* species (Gu et al. 2011a) and a crystal structure obtained (Gu et al. 2011b). A p-coumaric acid decarboxylase was purified from *Lactobacillus plantarum*. The enzyme was inducible and the purified enzyme had a K_M_ of 1.4 mM and a K_cat_ of 10^3^ s^−1^(Cavin et al. 1997b). The corresponding gene, *pdc*, was cloned and overexpressed in *E. coli* (Cavin et al. 1997a). This protein was crystallised and a structure obtained (Rodríguez et al. 2010). Based on the homology to the *fdc* and *pdc* a hydroxycinnamic acid (phenolic acid) decarboxylase, *pad*, was identified in *Bacillus subtilis* (Cavin et al. 1998). These hydroxycinnamic acid decarboxylases were shown to be active *in vivo* and *in vitro* and the purified enzymes did not require cofactors and were not part of multi subunit enzyme complexes. All these enzymes have in common that they are of bacterial origin and not active with cinnamic acid.

**Figure 1:**
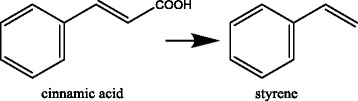
***Trans*** **-cinnamic acid is converted in a decarboxylation reaction to styrene.**

The yeast *Saccharomyces cerevisiae* shows some resistance toward cinnamic acid and the gene that confers this resistance was identified as the *PAD1* (phenylacrylic acid decarboxylase) gene (Clausen et al. 1994). The yeast can convert cinnamic acid to styrene (Figure 1) but also sorbic acid to 1,3-pentadiene however with a lower rate. The deletion of *PAD1* resulted in the inability to convert cinnamic or sorbic acid suggesting that the Pad1p is active with both substrates (Stratford et al. 2007). Overexpression of the *PAD1* gene resulted in strains with increased resistance to cinnamic and ferulic acid (Larsson et al. 2001). A *PAD1* homologue is also present in the mold *Aspergillus niger* where it confers resistance to cinnamic acid and sorbic acid (Plumridge et al. 2008). Recently it was shown in *S. cerevisiae* that not only the *PAD1* but the *PAD1* and the *FDC1* are essential for cinnamic acid, ferulic acid or coumaric acid resistance. Deletions in either of these two genes resulted in drastic reduced ability to decarboxylate these acids (Mukai et al. 2010). A possible interpretation of this observation was that the Pad1p and the Fdc1p are two essential subunits of a protein complex. The Pad1p and the Fdc1p are however believed to be in different cellular compartments. The Pad1p has a mitochondrial targeting sequence and the protein is located in the mitochondria (Huh et al. 2003) whereas the Fdc1p is a cytosolic protein.

The *PAD1* and the *FDC1* are clustered, i.e. they are located next to each other on the chromosomes. Such clusters with *PAD1* and *FDC1* homologues are widespread in yeast and filamentous fungi. In filamentous fungi the cluster contains also a transcription factor (Plumridge et al. 2010).

Cinnamic acid decarboxylase is required for the genetic engineering of styrene producing strains. Styrene can be produced from endogenous L-phenylalanine in two steps, by a phenylalanine ammonia lyase to produce cinnamic acid and a cinnamic acid decarboxylase producing styrene (McKenna and Nielsen 2011, McKenna et al. 2014). An efficient cinnamic acid decarboxylase would be desirable for the construction of strains for effective styrene production from biomass.

## Materials and methods

### Strains and plasmids

Strains: The yeast strain CEN.PK2-1C was obtained from Prof. Friedrich K. Zimmermann (Frankfurt) and used if not otherwise specified.

Plasmids: *PAD1* and *FDC1* were amplified from *S. cerevisiae* by PCR. The *PAD1* was amplified from genomic DNA of the yeast strain S288C (obtained from Dr. G. Shirleen Roeder, Yale), *FDC1* from the CEN.PK2-1C. Through PCR *Eco*RI and *Nhe*I sites were introduced and then ligated to the corresponding sites of p2159 and p2158. The p2159 was derived from the pYX212, a multicopy yeast expression vector with *URA3* marker and *TPI1* promoter, by changing the multiple cloning site as described before (Kuorelahti et al. 2005). The p2158 is derived in the same way from the pYX242, a multicopy yeast expression vector with *LEU2* marker and *TPI1* promoter. The *PAD1* was also amplified by PCR introducing *Xho*I and *Eco*RI sites and then ligated to the pFL60 (Minet et al. 1992), a multicopy yeast expression vector with *URA3* marker and *PGK1* promoter. The *PAD1* was also amplified by PCR without mitochondrial targeting signal. The mitochondrial targeting sequence was identified using the MitoProt software (http://ihg.gsf.de/ihg/mitoprot.html). In one case the first amino acid after the cleavage site was replaced by the start codon so that the amino acid sequence was MVAITG…. In another case the start codon was before the first amino acid resulting in the sequence MVVAITG…. An *FDC1* with mitochondrial targeting sequence was generated by adding the first 60 amino acids of the *PAD1* to the N-terminus of the *FDC1* open reading frame. The plasmids are listed in the Table 1.

**Table 1:** **Table of plasmids used**

**Plasmid name**	**Gene expressed**	**Parent plasmid**
p4237	FDC1	p2159 (URA3)
p4517	FDC1	p2158 (LEU2)
p4621	PAD1	pFL60 (URA3)
p4596	PAD1 without targeting (MVAITG…)	pFL60 (URA3)
p4597	PAD1 without targeting (MVVAITG…)	pFL60 (URA3)
p4787	FDC1 with mitochondrial targeting	p2158 (LEU2)

### Enzyme activity measurement

To prepare a cell free yeast extract yeast cells were collected by centrifugation, washed with water and 1 ml of fresh cell cake was suspended in 1 ml 20 mM sodium phosphate buffer pH 7.0. 1 ml of glass beads (0.4 mm diameter, Sigma) and protease inhibitor (Complete, Roche) were added and the cells were disrupted in two 40s sessions in the Fast Prep (Bio101). The cell extract was centrifuged and the supernatant analysed for cinnamic acid decarboxylase activity. The protein concentration was measured using the Biorad Protein Assay and BSA as a standard. The extract was added to a solution of cinnamic acid in 20 mM sodium phosphate, pH 7.0, and incubated at room temperature. The reaction was quenched by heating the sample to 96°C for 10 minutes.

Cinnamic acid and hydroxy-cinnamic acids were determined by using an analytical UPLC method. The separation of analytes was carried out on an Acquity UPLC BEH C18 1.7 μm column (2.1 × 100 mm) with a Waters Acquity UPLC system including sample manager-FTN, Quaternary solvent manager and PDA eλ detector. Detector was operated on 210-400 nm. The solvents used in gradient elution 0.43 mL/min were A) 5% formic acid and B) acetonitrile. Gradient system was as followed: 0 min 95% A and 5% B; 1.13 min 90% A and 10% B; 5.67 min 60% A and 40% B; 9.00 min 10% A and 90% B; and 10.00 min 90% A and 10% B. On the basis of corresponding standards (0-1000 μM) compounds were quantified.

### Analysis of styrene by SPME-GC/MS

Samples (300 μl) were analyzed by using SPME (solid phase micro extraction)-GC/MS. Extraction of styrene was done at 80°C for 30 min with preconditioned (300°C, 60 min) 75 μm Carboxen-PDMS fibre (Sulpelco, USA). After extraction the analytes were desorbed during 5 min at 250°C in the splitless injector (flow 14.9 ml/min) of the gas chromatography (Agilent 6890 Series; Palo Alto, CA, USA) combined with a MS detector (Agilent 5973Network MSD, USA) and SPME autosampler (Combipal, Varian Inc., USA). Analytes were separated on BPX5 capillary column of 60 m × 0.25 mm with a phase thickness 1.0 μm (SGE Analytical Science Pty Ltd, Australia). α-Pinene was used as internal standard. The temperature programme started at 60°C with 1 min holding time, then increased 7°C/min up to 100°C, followed by 10°C/min increase up to final temperature 200°C, where the temperature was kept for 4 min. MSD was operated in electron-impact mode at 70 eV, in the full scan m/z 40–550. The ion source temperature was 230°C and the interface was 280°C. Styrene was identified with corresponding standard and by comparing the mass spectra on Palisade Complete 600 K Mass Spectral Library (Palisade Mass Spectrometry, USA).

## Results

### *In vitro* activity

**Figure 2:**
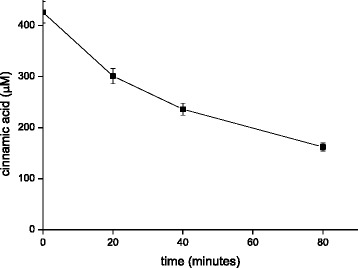
**Cinnamic acid decarboxylation with a cell free extract of a yeast strain over-expressing PAD1 and FDC.** The protein concentration is 0.32g/l.

It was previously shown that the *PAD1* is responsible for cinnamic acid dehydratase activity and that the overexpression of *PAD1* results in increased cinnamic acid dehydratase activity (Clausen et al. 1994, Larsson et al. 2001) using intact cells of *S. cerevisiae*. In order to test if this activity could be also seen in cell free extracts we analysed the cell free extracts of the CEN.PK strains which were not modified or expressed the *PAD1* gene. The *PAD1* gene was amplified from the *S. cerevisiae* strain S288C since the CEN.PK has a mutation in the *PAD1* gene resulting in a stop codon instead of the tyrosine in position 98. The cell extracts were incubated with 500 μM cinnamic acid at neutral pH and the disappearance of cinnamic acid was followed by UPLC, however no decarboxylase activity was detected. We followed the decrease in cinnamic acid over a period of 5 hours at a protein concentration of 2 g/l but could not detect a decrease. The detection limit for the decrease in concentration was estimated 15 μM. We also tested if the overexpression of *FDC1* would result cinnamic acid decarboxylase activity in the cell free extract however no activity was detected. However when the two cell-free extracts with *PAD1* and the extracts with *FDC1* were mixed, cinnamic acid decarboxylase activity was detected. After 5 hours the cinnamic acid concentration was decreased by 250 μM. This suggested that both enzymes, Pad1p and Fdc1p, are required. We then expressed *PAD1* and *FDC1* in the same yeast strain using two multicopy plasmids. This extract had a much higher cinnamic acid decarboxylase activity. This is summarized in the Figure 2. The initial rate was about 0,019 μmol per minute and mg of extracted protein. We also confirmed that the reaction product was styrene.

### *In vivo* activity

The *PAD1* gene has a mitochondrial targeting sequence of 57 amino acids and the Pad1p is believed to be a mitochondrial protein. The *FDC1* gene however has no targeting sequence and the Fdc1p is believed to be a cytosolic protein. To test if Pad1p and Fdc1p are also active *in vivo* we tested the sensitivity toward cinnamic acid. For that purpose we plated the yeast strain over-expressing the *PAD1* and *FDC1* on selective plates with increasing amounts of cinnamic acid. At concentrations of up to 10 mM cinnamic acid growth was observed. In the control concentrations above 100 μM were toxic (Table 2). The pKa for cinnamic acid is 4.44. Since the medium was not buffered, the cinnamic acid is in its salt form and it is actually cinnamate resistance that was observed.

**Table 2:** **Growth on increasing cinnamic acid concentrations**

**Cinnamic acid concentration**	**H4086**	**Control**
30 mM	-	-
20 mM	-	-
10 mM	+	-
5 mM	+	-
3 mM	+	-
1 mM	+	-
500 μM	+	-
100 μM	+	+

In the strain H4086 PAD1 and FDC1 are expressed from two multicopy plasmids. The control strain harbours the plasmids without open reading frames.

### Mitochondrial targeting

The combination of *PAD1* and *FDC1* seemed to result in cinnamic acid decarboxylase activity although the proteins are targeted to different cellular compartments. We tested if the activity could be increased by targeting the proteins to the same cellular compartment. In one case we targeted both proteins to the mitochondria. For that purpose we added the mitochondrial targeting sequence of the *PAD1* to the *FDC1*. In the other case we targeted both to the cytosol. For that we removed the targeting signal from the *PAD1*. In both cases the cell free extract of the yeast strain over-expressing both genes, *PAD1* and *FDC1* with added targeting sequence or *FDC1* and *PAD1* with removed targeting sequence, showed no cinnamic acid decarboxylase activity. Removing or adding mitochondrial targeting signals might affect the protein folding, the lack of activity is therefore not necessarily related to the targeting.

### Activity with hydroxy-cinnamic acids

**Figure 3:**
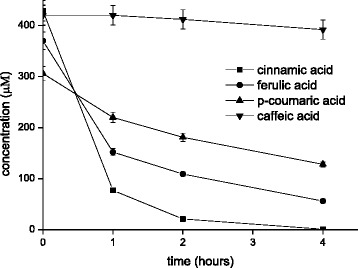
**Decarboxylation of the hydroxy cinnamic acids: ferulic acid, p-coumaric acid and caffeic acid.** The protein concentration is here 1.6 g/l.

We tested the activity with the following hydroxyl-cinnamic acids in cell free extracts: p-coumaric acid, ferulic acid and caffeic acid. When the *PAD1* or *FDC1* were over-expressed alone we could not detect decarboxylase activity. However, when overexpressing *PAD1* and *FDC1* together we observed activity. This is summarized in the Figure 3. The highest activity was observed with ferulic acid, but this activity was still lower that with cinnamic acid.

## Discussion

Although it is known that yeast and other microorganisms have the ability to convert cinnamic acid to styrene, the enzyme to catalyse this reaction has not been identified. This is mainly because most of the studies were carried in vivo and only a few used a cell free environment (Mukai et al. 2010, Stratford et al. 2012).

In *S. cerevisiae* the *PAD1* was suggested to code for the cinnamic acid decarboxylase. A *PAD1* mutant showed increased sensitivity toward cinnamic acid and expression of *PAD1* repaired the phenotype (Clausen et al. 1994). In the same study permeabilised cells were used to measure cinnamic acid decarboxylase activity. The parent strain showed an activity of 0–3.9 nM/mg dry mass per h and after expression of PAD1 29.4 nM/mg dry mass per h (Clausen et al. 1994). Assuming that 40% of the dry mass is extractable protein this would correspond to about 75 nM per mg extracted protein and hour. When we overexpressed *FDC1* and *PAD1* and analysed the cell extract we estimated the decarboxylase activity to be about 1 mM per mg extracted protein and hour. This corresponds to an increase of activity of about 4 orders of magnitude.

The Pad1p and the Fdc1p are located in different compartments in *S. cerevisiae*. The Pad1p is in mitochondria and the Fdc1p in the cytosol. The fact that the overexpression of both proteins results in an increased resistance toward cinnamic acid can be interpreted in different ways. One interpretation is that cinnamic acid is converted to styrene in two steps with an intermediate that is passing through the mitochondrial membrane. Another interpretation is that the proteins are not confined to their compartment. However for the Pad1p it was shown that after tagging with the green fluorescence protein the enzyme was exclusively located in the mitochondria (Huh et al. 2003).

No information is available about a possible protein complex including Pad1p and Fdc1p. In an genome wide survey to identify protein complexes by systematic tagging of open reading frames to identify protein complexes, no protein complexes with Pad1p or Fdc1p were identified (Gavin et al. 2006).

The physiological role of cinnamic acid decarboxylation is detoxification. Cinnamic acid or cinnamate is more toxic than the reaction product styrene. Cinnamic acid decarboxylation is also of potential biotechnological relevance. L-Phenylalanine can be efficiently converted to cinnamic acid by the action of an L-phenylalanine ammonia lyase (PAL). PAL and cinnamic acid decarboxylase constitute a pathway for styrene production from L-phenylalanine. This had been practiced by McKenna et al. in *E. coli* to produce styrene from D-glucose (McKenna and Nielsen, 2011). In this work the *PAD1* and the *FDC1* of *S. cerevisiae* were expressed in *E. coli* but it was concluded that functional cinnamic acid decarboxylase activity in *E. coli* depends solely upon *FDC1* over-expression and is not dependent on co-expression of *PAD1*. The expression of the *S.cerevisiae PAD1* in *E. coli* did not result in cinnamic acid decarboxylation activity; however the combined expression of *PAD1* and *FDC1* resulted in such activity. The expression of FDC1 alone also resulted in activity suggesting that the *PAD1* is not required for activity; however it cannot be excluded that an *E. coli* protein homologous to *PAD1* would be functional (McKenna and Nielsen, 2011). There are indeed proteins in *E. coli* with homologies to the *PAD1*. One is the *UbiX* that has a role in coenzyme Q biosynthesis (Gulmezian et al. 2007). Another one is a close homologue, *Pad1*, that has been crystallized and a structure determined (Rangarajan et al. 2004), however no function was assigned.
